# Suicides in Barnsley – an IHBTT project

**DOI:** 10.1192/bjo.2021.860

**Published:** 2021-06-18

**Authors:** Nadia Imran, Omair Niaz

**Affiliations:** Intensive Home Based Treatment Team

## Abstract

**Aims:**

We wanted to see whether an increase in IHBTT(Intensive Home based treatment team) case load correlated with the recent increase in suicides. We also wanted to investigate the common factors between patients who died by suicide.

**Background:**

This was a study completed by IHBTT in Barnsley (South Yorkshire), looking into recent suicides with the caseload from April 2009 to November 2019. There were a total of six suicides.

**Method:**

We Calculated mean IHBTT caseload size from November 2008 to November 2019 . There were 6 suicides in this period. We plotted this against caseload, investigating if increase in caseload correlated with these. We also analysed the common themes and trends associates with these patients who died by suicide. We compared the trends we found locally against a National Survey. (National Confidential Inquiry into Suicide and Safety in Mental Health; Annual Report: England, Northern Ireland, Scotland, Wales October 2018 University of Manchester).

**Result:**

We found that four out of six suicides occurred during periods of high activity.. Common themes we found around patients who had died by suicide included middle aged men who lived alone, with a diagnosis of adjustment disorder, recent financial stress and relationship breakdown, upcoming court case, abusing drugs or alcohol. This does compare somewhat to national trends, however alcohol and drug misuse, upcoming court case and financial stressors and relationship breakdown are higher in our patients who died by suicide compared to nationally.

**Conclusion:**

We acknowledge the small sample size and hence the need to take results cautiously. However there is a clear increase in suicides as caseload increases, we hypothesised this was due to the same levels of staff despite increase in caseload. We were also able to conclude the factors our patients who died by suicide had in common locally, and how this compared to national data. We wondered if this could be used to guide resource allocation, i.e. interventions to help patient manage their finances, accommodation and substance misuse. Consideration may need to be given to reviewing IHBTT staffing levels, given the significant decrease in inpatient bed numbers.

